# DACBT: deep learning approach for classification of brain tumors using MRI data in IoT healthcare environment

**DOI:** 10.1038/s41598-022-19465-1

**Published:** 2022-09-12

**Authors:** Amin ul Haq, Jian Ping Li, Shakir Khan, Mohammed Ali Alshara, Reemiah Muneer Alotaibi, CobbinahBernard Mawuli

**Affiliations:** 1 School of Computer Science and Engineering, University of Electronic Science and Technology of China, Chengdu, 611731 China; 2 College of Computer and Information Sciences, Imam Mohammad Ibn Saud Islamic University, Riyadh, 11432 Saudi Arabia

**Keywords:** Computational biology and bioinformatics, Diseases, Engineering, Mathematics and computing

## Abstract

The classification of brain tumors (BT) is significantly essential for the diagnosis of Brian cancer (BC) in IoT-healthcare systems. Artificial intelligence (AI) techniques based on Computer aided diagnostic systems (CADS) are mostly used for the accurate detection of brain cancer. However, due to the inaccuracy of artificial diagnostic systems, medical professionals are not effectively incorporating them into the diagnosis process of Brain Cancer. In this research study, we proposed a robust brain tumor classification method using Deep Learning (DL) techniques to address the lack of accuracy issue in existing artificial diagnosis systems. In the design of the proposed approach, an improved convolution neural network (CNN) is used to classify brain tumors employing brain magnetic resonance (MR) image data. The model classification performance has improved by incorporating data augmentation and transfer learning methods. The results confirmed that the model obtained high accuracy compared to the baseline models. Based on high predictive results we suggest the proposed model for brain cancer diagnosis in IoT-healthcare systems.

## Introduction

Brain tumor (BT) is a series medical problem, and many people are suffering from it globally^1^. Because of its critical nature, brain tumours are one of the most dangerous types of brain cancer. Compared to other cancers from brain cancer less number of people are suffering^2^. Meningioma, Glioma, Pituitary, and Acoustic Neuroma are examples of brain tumors. In medical observation, the rates of Meningioma, GLioma, and Pituitary tumours in all brain tumors are 15%, 45%, and 15%, respectively^3^. A brain tumor has long-term and psychological consequences for the patient. Brain tumors are caused by tissue abnormalities that develop within the brain or the central spine, interfering with normal brain function. There are two types of brain tumors: benign and malignant. Benign brain tumors are not cancerous and grow slowly. They do not spread and are not common. Malignant brain tumours contain cancer cells and grow rapidly in one region of the brain and spread to other parts of the brain and spine.

The diagnosis of brain cancer is significantly necessary for early stage for effective treatment and recovery. In this regards to classify brain tumors and identify brain cancer, different non-invasive are developed in literature by researchers and medical experts in Internet of Things (IoT) healthcare industries. In the deigning of computer automatic diagnostic systems (CADS) for brain cancer detection Machine Learning (ML) and Deep Learning (DL) models are commonly used. The diagnosis of brain cancer using images data using the DL Convolution neural network (CNN) model has grown in popularity, and the CNN model is commonly used for image classification and analysis, particularly for medical image data analysis^4^. The CNNs model can extract more related features from data for accurate image classification^2,5,6^. Furthermore, data augmentation and transfer learning techniques can also improve the predictive capability of deep learning models to effective classify the brain tumors and diagnosis brain cancer in IoT healthcare industries^6,7^.

In the literature, various methods have been proposed for brain cancer diagnosis using ML and DL learning approaches by different scholars. Zacharaki et al.^8^ designed a brain cancer diagnosis system to classify various grades of Glioma employing SVM and KNN machine learning model and respectively achieved 85% and 88% classification accuracy. Cheng et al.^9^ proposed a classification approach for brain tumor classification and augmented the tumor region for improving the classification performance. They employed three techniques for feature extraction such as Gray level co-occurrence matrix, a bag of words, and an intensity histogram. Their proposed method obtained 91.28% classification accuracy.

Haq et al.^6^ proposes an AI-based intelligent integrated framework (CNN-LSTM) for brain tumors classification and diagnosis in the IoT healthcare industry. In the integrated framework design, they have incorporated the CNN model to extract features from medical MRI data automatically. The extracted features are passed to Long short-term memory (LSTM) model to learn the dependencies in the features and finally predict the class for the tumor. Further they applied brain MRI data sets for the assessment of the proposed integrated model. Massive data is one requirement for an effective deep learning model. Since the size of our original data set is small, they utilized data augmentation approaches to increase the data set size, thereby improving the model result during training. Also used the train-test splits Cross-validation approach for hyperparameter tuning and best model selection to ensure proper model fitting. For model assessment, used well-known evaluation measures. They compared the predictive outputs of the proposed CNN-LSTM model with previous methods in the Medical Internet of Things (MIoT) healthcare industry and the model obtained high predictive performance.

Paul et al.^4^ employed axial brain tumor images for convolution neural network training. In the proposed method they used two convolution layers, two max-pooling layers, and lastly, two fully connected layers for the final classification process. The proposed approach obtained 91.43% classification accuracy. El-dahshan et al.^10^ designed a brain tumors classification method for 80 brain images MRI classification. They used discrete wavelet transform and PCA algorithms for reducing dimensions of data. To classify the normal and abnormal tumors, they used ANN and KNN machine learning classifiers. The classifiers ANN and KNN, achieved 97% and 98% classification accuracy respectively.

In another study, Afshar et al.^11^ proposed a brain tumor classification method employing a capsule network that combined MRI images of the brain and coarse tumor boundaries and 90.89% accuracy achieved by the proposed method. Anaraki et al.^12^ developed an integrated framework for brain tumor classification, and in the proposed technique, they integrated CNN and GA, and designed GA-CNN framework and obtained 94.2% accuracy. Khan et al.^13^ proposed brain tumors classification method employing transfer learning techniques (CNN-Transfer learning) and achieved 94.82% accuracy^14^. The proposed multi-classification method employing ensemble of deep features and ML algorithms and obtained high performance.

According to the review of the literature, current brain cancer diagnosis techniques still lack a robust predictive capability in terms of accuracy to correctly diagnose brain cancer for proper treatment and recovery. To address this issue, a novel robust method for accurately diagnosing brain cancer for proper treatment and recovery in IoT healthcare industries is required. Furthermore, the artificial intelligence based brain cancer diagnosis systems also reduce the financial costs of healthcare department.

In this study, we created an improved CNN model for the classification of brain MR images to diagnosis brain cancer in IoT healthcare industries. In the development of the proposed model, we used Convolution neural network model to classify brain tumors types (Meningioma, Glioma and Pituitary) employing MR images data. The CNN model is more suitable for the Meningioma, Glioma, and pituitary classification using brain tumors images data and its extract more deep features from images data for final classification. To further improve the CNN model predictive capability, we have incorporated a transfer-learning (TL) techniques for proper training of the CNN architecture, the brain MR images data is insufficient. In transfer learning, we used the well-known pre-trained models ResNet-50, VGG-16, Inception V3, DenseNet201, Xception, and MobilleNet. The weights generated of these pre trained models individually transferred to CNN architecture for effective training o CNN model. For the fine-tuning process, the model was trained with brain MR images data set. The generated weights of pre trained models improving CNN model final predictive performance. Additionally, the data augmentation technique is incorporated to increase the data set size for effective training of the model. We also used held-out cross-validation (CV) and performance evaluation metrics. The performance of the model compared with base lines models. The experimental results confirmed that the proposed model generated higher predictive results and it could be applied in IoT-healthcare systems easily.

Innovations of this study summarized as follows:

- In IoT healthcare systems, an improved model based on CNN and TL for classifying brain tumors using MR image data is proposed for diagnosis of brain cancer.
- To increase the predictive accuracy of the CNN model, TL techniques are used because the brain tumor image data is insufficient for effective training of the CNN model. Pre-trained models ResNet-50, VGG-16, Inception V3, DenseNet201, Xception, and MobilleNet are used to train with the well-known ImageNet data set for generating trained parameters (weights). The weights of these pre tained models are individually transfer to CNN model effective training. Fine-tuning the model CNN with brain tumor images data along with transferred weights final classification.
- To improve model performance, the data augmentation technique is used to increase the size of the data set for effective model training.
- When compared to baseline methods, our model has a high predictive performance.

The rest of the paper is organized as follows: In “Materials and method” section data set and proposed model methodology have explored. In “Experiments” section the experiments are reported. In “Discussion” section, we discussed the significance of the work. The conclusion and research direction of future work are reported in “Conclusion” section.

## Materials and method

### Data set

We used a brain tumor data set (BTDS) from China’s Nanfang hospital and general hospital, as well as Tianjing medical university, in this study (2005 to 2010)^9^, and new versions in 2017 have been published. T1-Weighted Contrast-Enhanced images (TWCEI) of 233 subjects with meningioma, glioma, and pituitary tumours are included in this data set. The data set is freely accessible via the Kaggle repository^15^. We also used the Brain MRI Images Data Set (BMIDS) for cross dataset validation, which contains 253 MRI brain images. The tumor class in the data set has 155 images, while the non-tumor class has 98 images^16^.

### Background of convolutional neural network (CNN) architecture

Deep Learning model convolutional neural networks is a kind of Feed-Forward Neural Network^17^. Convolutions can capture translation invariance, which means that the filter is independent of position that significantly reduces the number of parameters. The CNN model have Convolutional, Pooling, and fully connected layers. Different functions are accomplished by these layers, such as dimensionality reduction, feature extractors, and classification. During the convolution operation of the forward pass, the filter is slide on the input shape and compute the map of activation, which computing the point-wise value of each output. Further add these output to achieve the activation of that point. Designed a Sliding Filter (SF) using convolution as a linear operator, and expressed as a dot product for fast deployment. Let consider *x* and *w* are input and the kernel function, the convolution process $(x*w)(a)$ on time index *t* can be mathematically expressed in Eq. (1).

1 $$\begin{aligned} (x*w) a=\int x(t) w (a-t)da \end{aligned}$$

In Eq. (1) *a* is in $\text{ R}^n$ for any $n \ge 1$. While Parameter *t* is discrete. In this case, the discrete convolution can be expressed as in Eq. (2):

2 $$\begin{aligned} x.w (a) = \sum _{a}x w (t-a) \end{aligned}$$

However, usually use 2 or 3-dimensional convolutions in CNN model. In case of 2-dimensional image *I* as input and *K* is a two dimensional kernel and the convolution can be mathematically expressed as in Eq. (3):

3 $$\begin{aligned} (I*K)(i,j)=\sum _{m}\sum _{n}I(m,n)K(i-m,j-n) \end{aligned}$$

If the case is 3 dimensional data image, then the convolution process can be written mathematically in Eq. (4) as follow:

4 $$\begin{aligned} (I*K)(i,j,k)=\sum _{m}\sum _{n}\sum _{l}I(m,n,l)K(i-m,j-n,k-l) \end{aligned}$$

In addition to gain non-linearities, two activation functions can be incorporate suc as Sigmoid and ReLU. The sigmoid activation fumction non-linearity is expressed mathematically in Eq. (5):

5 $$\begin{aligned} \theta (x)=\frac{1}{1+exp(-x)}, x \in R. \end{aligned}$$

The sigmoid non-linearity activation function is suitable when need the output to be include in the range of [0,1]. Furthermore, the sigmoid function is monotone growing which means $\lim \limits _{n \rightarrow +\infty } \theta (x)=1$, and $\lim \limits _{n \rightarrow +\infty } \theta (x)=0$. However, this fact may be cause vanishing gradients, when the input *x* is not near to 0, the neuron will be more and the gradient of $\theta (x)$ will nearly to zero and will make successive optimization difficult.

The second activation function is relu which is mathematically defined in Eq. (6):

6 $$\begin{aligned} Relu(x) = max(0, x), x \in R \end{aligned}$$

The gradient of of $relu(x)=1$ for $x>0$ and $relu^-(x)=0$ for $x<0$. The relu convergence capability of is good then sigmoid non-linearities.

The CNN model Pooling layers are utilized to produce a statistics summary of its inputs and deduced the dimensionality without missing important information. There are different types of pooling. In the layer of Max-Pooling generate the extreme values in individually rectangular neighborhood of individual point i.e i, j, k for data of three dimensional of individual feature of input respectively, while the average values generated by the average pooling layer.

The last layer is fully connected with *n* and *m* respectively input and output sizes. The output layer is expressed by the parameters such as a weight matrix i.e $W \in M_{m, n}$ with *m* rows, and *n* columns and a bias vector $b \in {\textbf {R}}^m$. The input vector $x \in {\textbf {R}}^n$, the fully connected output layer *FC* along function of activation *f* is expressed mathematically in Eq. (7) as:

7 $$\begin{aligned} FC(x): = f (Wx+b) \in R^m \end{aligned}$$

In Eq. (7) *Wx* is the product matrix while the function *f* is used component wise.

The last layers fully connected employed for classification of problems. The CNN model architecture last layer is fully connected layers and CNN output is flattened and showed as a single vector.

### Convolution neural network for brain tumors classification

Recently, CNN models generated significant outcomes in numerous domains, such as NLP, image classification^18^, and diagnosis systems. In contrast to MLPs, CNN reduces the number of neurons and parameters, which results in lower complexity and faster adaptation.

**Figure 1:**
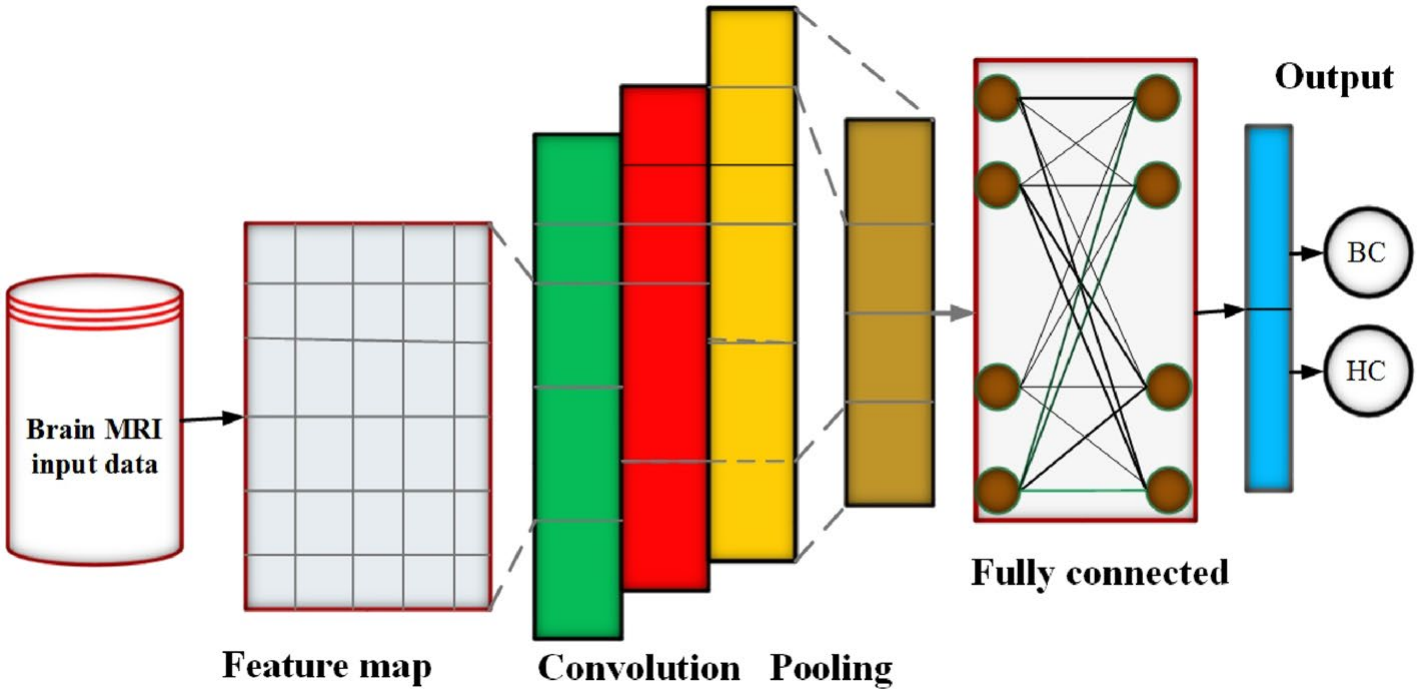
CNN model architecture for classification of Brain tumors.

The CNN model has significant applications in the classification of medical images^18,19^. In this paper we developed the CNN networks architecture with 4 alternating convolutional layers and max-pooling layers and a dropout layer after each Conv/pooling pair. The last pooling layer connected fully layer with 256 neurons, ReLU activation function, dropout layer, and sigmoid activation function are employed for classification of brain MR images (Meningioma, Glioma, and Pituitary). In addition, we have used the optimization algorithm Stochastic Gradient Descend (SGD)^20^. The CNN architecture is given in Fig. 1.

### Improve CNN model for brain tumors classification

To improve CNN model predictive accuracy, we employed Data augmentation (DA) and Transfer learning (TL) techniques. The data augmentation can resolve the problem of insufficient data for model training. To expand the data amount, the zooming technique is used on original image data to produce images data with the similar label. The new created data set is used for fine tuning of the model. Th The transfer learning (TL) techniques widely used in image classification tasks^21^, cancer sub-type recognition^22^ and medical images filtering^23^. In this work, we used the transfer learning ResNet-50, VGG-16, Inception V3, DenseNet201, Xception, and MobilleNet models to enhanced the predictive performance of the proposed CNN model. The ResNet-50, VGG-16, Inception V3, DenseNet201, Xception, and MobilleNet pre-train models were trained on imageNet data set and transferred the trained parameters weights of these models individually to CNN model for effective training, and fine-tuned the model using the brain tumor augmented MR images data set for final classification of the CNN model.

### Model cross validation and evaluation criteria

The holdout cross-validation^6,24,25^ mechanism was used for training and validation of the model. In hold out CV data is randomly assign to two sets $d_0$ and $d_1$. The $d_0$ and $d_1$ use for training and testing of the model respectively. In hold out CV the training data set is usually large as compare to testing data set. The is train on $d_0$ and testing on $d_1$. The holdout CV is suitable validation method in case when the data set is very plenty. In this study brain tumor MRI Images data set was divided into 70% for training and $30\%$ for teasing of the model. The performance evaluation metrics Accuracy (Acc), Sensitivity (Sn), Specificity (Sp), Precision (Pr), F1-Score (F1-S), and Matthews Correlation Coefficient (MCC)^26–29^ are used for model evaluation.

### Proposed brain tumors classification model

NCNN models are now popular for image classification problems. A large image data set is more suitable for the CNN model’s effective training, as it allows the model to extract more related features during the training process for accurate image classification. The CNN model’s performance suffers as a result of the scarcity of large image data sets, particularly in the medical domain. However, to enhance the proposed CNN classifier performance, data augmentation and transfer learning^6,21,30,31^ techniques are incorporated. We have used transfer learning pre-trained models ResNet-50, VGG-16, Inception V3, DenseNet201, Xception, and MobilleNet along with data augmentation technique zooming. The imagesNet data set has been employed for pre-trained of ResNet-50, VGG-16, Inception V3, DenseNet201, Xception, and MobilleNet models, and the generated weights (trained parameters) of these models were transferred for the effective training of the CNN model individually. Brain tumor MRI data set was used for fine-tuning of CNN model and for final classification of the model in IoT healthcare system.

Furthermore, the proposed CNN model was trained and tested on a data set of brain tumour MR images, and its performance was compared to that of the transfer learning technique. A heldout cross-validation mechanism is used in the proposed method for model training and testing, with 70% used for training and 30% for model validation. The data augmentation^20^ technique was used to augment the original dataset by using the zooming method, which improves the model generalisation capability. The integration of data augmentation and transfer learning greatly enhanced the predictive accuracy of the CNN model. The evaluation criteria of the model different assessment metrics have used.

**Figure 2:**
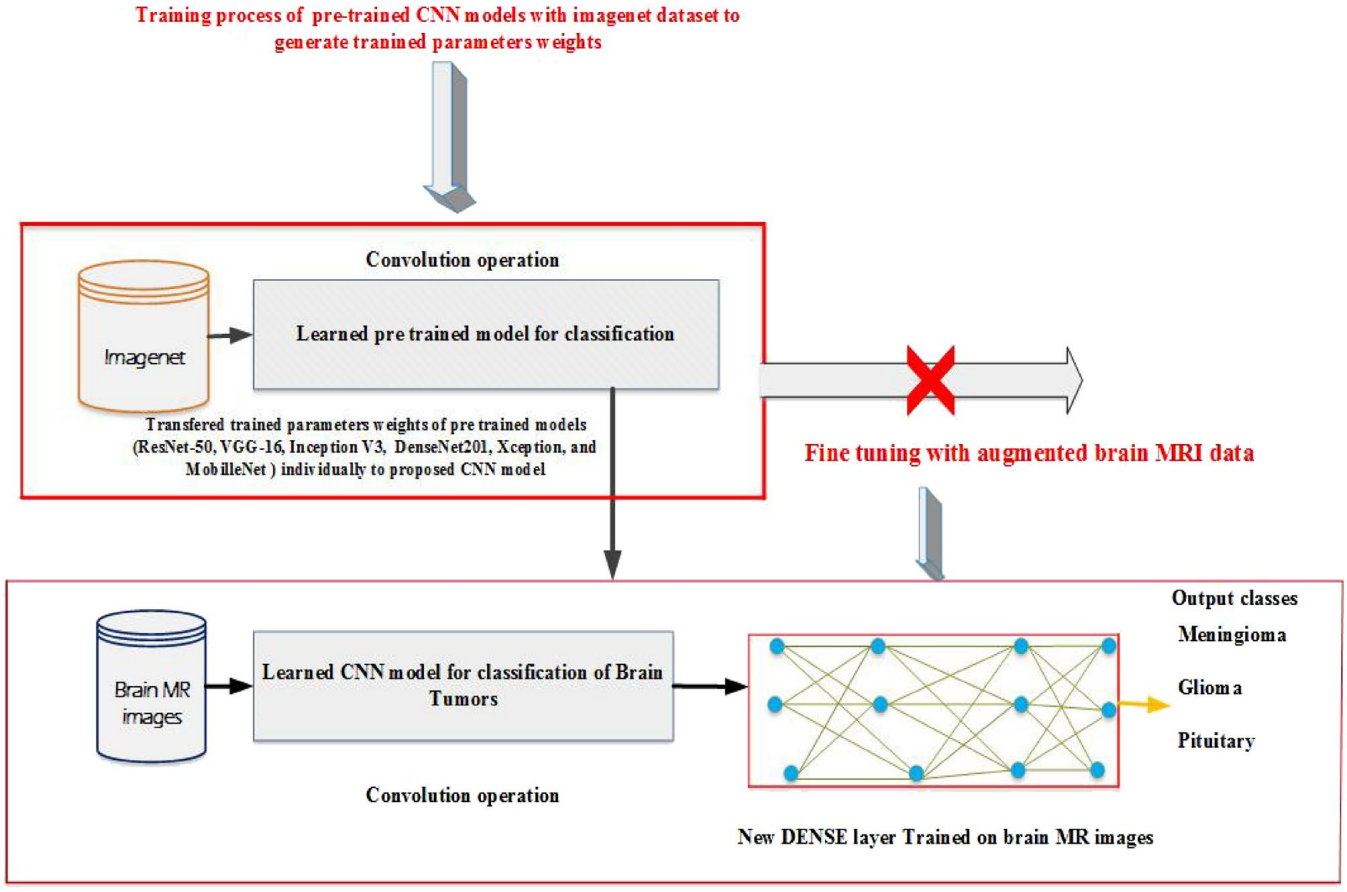
Flow chart of proposed tumor classification framework in IoT healthcare systems. The pre trained CNN models (ResNet-50, VGG-16, Inception V3, DenseNet201, Xception, and MobilleNet) are trained with image-net dataset and the generated weights of these pre trained models are individually transferred to proposed CNN model for effective training. While the augmented data set is used for fine-tuning of the ResNet-CNN model for final classification of brain tumors.

The data set *X*(*i*, *i*) embedded into the CNN classifier,We used data transformations to increase the size of the data set so that we could train the model. Furthermore, the number of epochs *E*, model parameters *w*, Learning Rate (LR) $\eta$, size of batch *b*, and the number of layers in both CNN were configured accordingly. For the optimization of our model parameters, we have used the stochastic gradient descent algorithm (SGD). The pseudo-code of the proposed model is given in algorithm 1 and flow chart in Fig. 2.
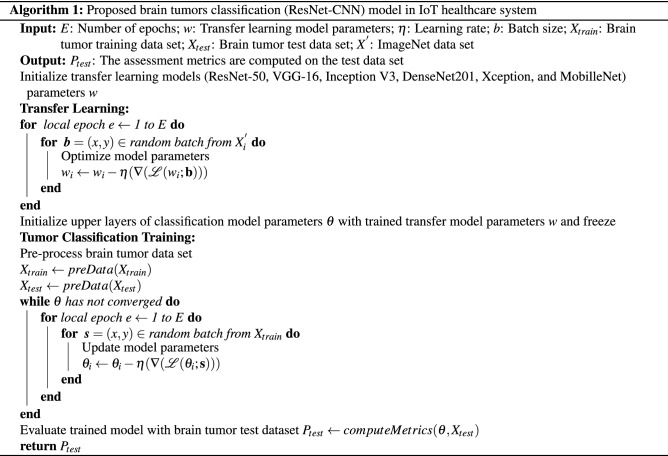

## Experiments

### Experimental setup

We conducted various experiments to test the feasibility of our proposed model in IoT healthcare system. The proposed model was tested using a brain tumour image data set in this study. To improve the proposed CNN model predictive performance, we have employed (ResNet-50, VGG-16, Inception V3, DenseNet201, Xception, and MobilleNet) CNN pre trained models with imagenet dataset to produce high trained parameters (weights) and then transferred trained parameters weights of pre trained models to the CNN model individually for effective training of the model. For fine-tuning the CNN model, the brain tumor images data set was employed for final classification. The brain tumor data have 233 subjects and 3064 slices, which belong to three classes, i.e., Meningioma, Glioma, and Pituitary. This data set is very Small for effective training of the CNN model. In addition to tackle the problem of small brain tumor data method of data augmentation^20^ has used to augment the original data set. Data augmentation technique (zooming) is used, and all three types of images (Meningioma, Glioma, and Pituitary) are zoomed horizontally and vertically and added with existing images. The new augmented data set image size of three kinds of images is 6128. Held out technique is used for model training and validation, and respectively 70% and 30% data are employed for training and validation of the model for all experiments. To effectively optimize the model SGD Optimization algorithm is used^20^. In addition, other parameters such as learning rate (LR) $\alpha$, SGD = 0.0001, epochs = 100, batch size = 120, outer and inner activation function = ReLu is used in all experiments. It is worth noting that for the final prediction layer our CNN model, the softmax activation function was used. Evaluation metrics are incorporated to evaluate the model performance.

All experiments used a laptop and a Google collaborator with GPU. All experiments required Python v3.7, and the CNN model was created using Keras framework v2.2.4 as a high-level API and Tensor flow v1.12 as the back end. All experiments were repeated numerous times to obtain consistent results. All experiment results were tabulated and graphed.

### Results and analysis

#### Results of data pre-processing

The brain tumor data set (BTDS) is obtained from the Kaggle repository^15^. T1-weighted contrast-enhanced images of 233 meningioma, glioma, and pituitary tumour patients are included in this data set. The Brain Tumor data contains 233 subjects and 3064 slices, with meningioma subjects accounting for 82 with slices 708, glioma subjects accounting for 91 with slices 1426, and pituitary subjects accounting for 60 with slices 930. Thus, the total number of subjects in the data is 233, and the total number of slices is 3064. In order to reduce the dimension of $512\times 512\times 1$ into $224\times 224\times 1$ for effective training of model.

**Figure 3:**
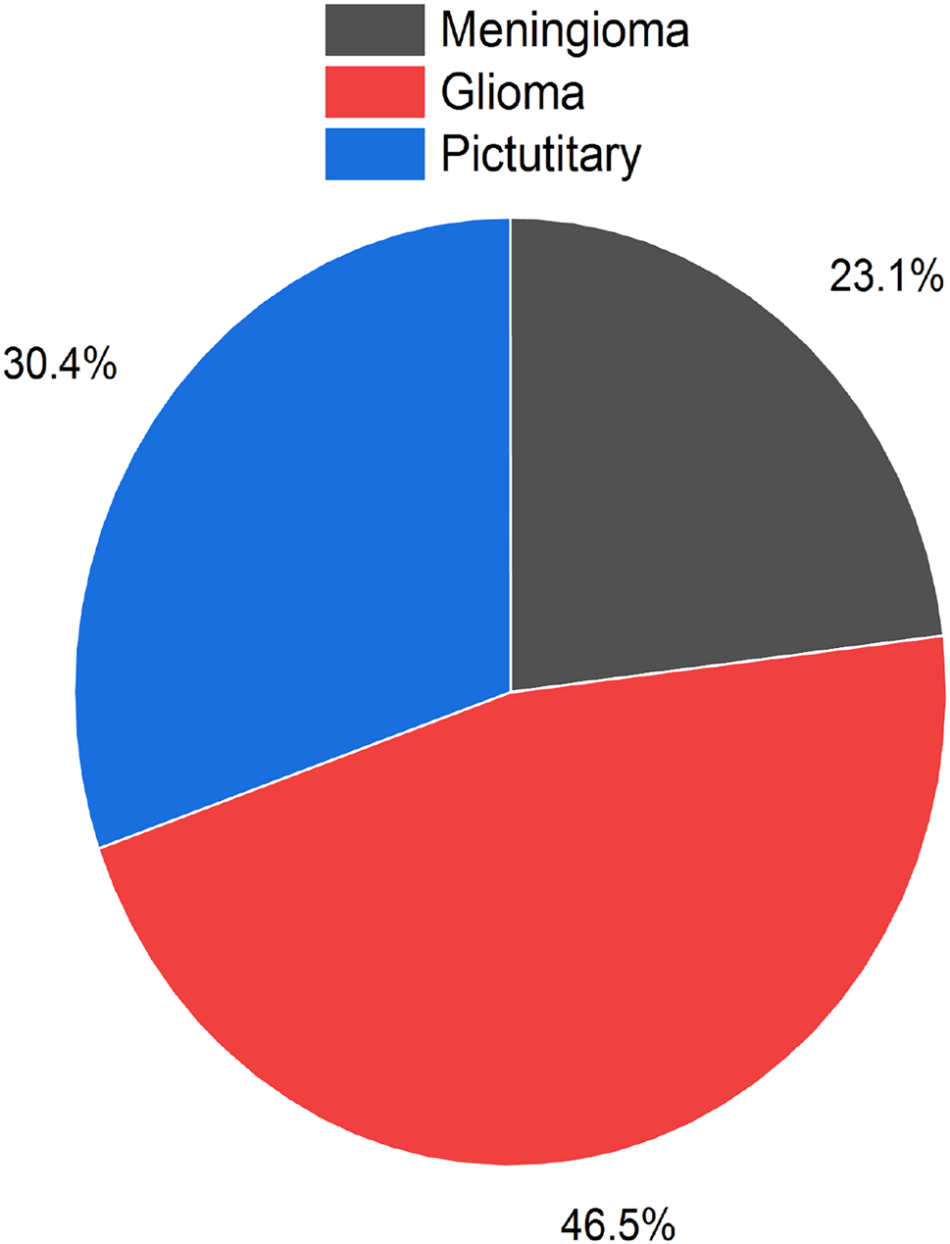
Ratio of samples in data set.

To handle imbalance problem in data set because Brain tumor data set has the different number of three subjects slices. The distribution of the data is different, and it creates a problem of over fitting the model. To balance the meningioma, glioma, and pictutitary in the data set, we incorporate the data augmentation^20^ method to augment the original dataset by using random zooming. All slices are being zoomed, and a new data set with 6128 slices has been created. The ratio of samples in an original data set is shown in Fig. 3. The data set has three subfolders for meningioma, glioma, and pictutitary images. Held out techniques is used for model training and validation because the new data set is very big and heldout validation is suitable in case of plenty dataset. The data set has splitted into 70% and 30% for training and validation of the model respectively. The cross-validation method has also been employed for an augmented data set.

We also used the Brain MRI Images Data Set (BMIDS) for cross dataset validation, which contains 253 MRI brain images. The tumor class in the data set has 155 images, while the non-tumor class has 98 images.

#### Results of the proposed CNN model, on original and augmented data sets

The performance of the proposed CNN model is evaluated using the original and augmented brain tumour MR image data sets. The CNN model is configured with essential hyper-parameters such as optimizer SGD with a Learning Rate (LR) of 00.0001, epochs 100, and size of batch was 120. The 70% data for training and 30% for the testing of the model is used. Different evaluation matrices were used for model performance evaluation. The input image size $264\times 264\times 1$ is used for training and evaluation of the proposed CNN model. All these hyper-parameters values and the output of the experimental results have been reported in Table 1.

Table 1 presented the proposed CNN model obtained 97.40% accuracy, 98.03% specificity, 95.10% sensitivity, 99.02% Precision, 97.75% MCC, and 97.26% F1-score respectively on original brain tumor MR images data set. The 97.40% accuracy demonstrated that our CNN architecture accurately classifies the three classes of brain tumors (meningioma, glioma, and pictutitary). The 98.03% specificity shows that the Proposed CNN model is a highly suitable detecting model for healthy subjects recognition, while 95.10% sensitivity presents that the model significantly detected the affected subjects. The MCC value was 97.75%, which gives confusion metrics a good summary.

On the other hand, the CNN model gained very excellent performance when trained and evaluated on an augmented data set. The CNN model obtained 98.56% accuracy, 100.00% specificity, 98.09% sensitivity, and 98.00% MCC when trained and evaluated on an augmented data set. The accuracy of the model improved from 97.40 to 98.56% which demonstrated the importance of the data augmentation process. Also, it illustrated that model needs more data for effective training of the CNN model.

From the experimental results, we concluded that the proposed CNN model effectively classified the brain tumor types, and the augmentation process further improved the model CNN performance because the CNN model more data for extract more related features for classification. The high accuracy of the proposed CNN model might be due to the suitable architecture of the CNN model and proper fitting of essential parameters of the model and data augmentation.

**Table 1:** CNN model performance on original and augmented data sets.

Data set	Parameters		Assessment metrics					
	Optimizer	LR	Acc (%)	Sp (%)	Sn (%)	Pr (%)	MCC (%)	F1-S (%)
Original	SGD	0.0001	97.40	98.03	95.10	99.02	97.75	97.26
Augmented	–	–	98.56	100.00	98.09	97.12	98.00	98.10

#### CNN model performance evaluation with cross dataset

We have evaluated the predictive performance of CNN model with independent cross dataset. We trained the proposed CNN model with original and augmented brain tumor data set and validated with independent Brain MRI Images Data Set (BMIDS). The model is configured with essential hyper-parameters such as optimizer SGD with a Learning Rate (LR) of 00.0001, epochs 100, and size of batch was 120. Different evaluation matrices were used for model performance evaluation. The input image size $264\times 264\times 1$ is used for training and evaluation of the proposed CNN model. The experimental results of model with cross data are reported in Table 2.

Table 2 presented that the proposed CNN model obtained 97.96% accuracy, 99.00% specificity, 97.30% sensitivity, 98.18% Precision, 98.00% MCC, and 99.02% F1-score when trained on original brain tumor MR images data set (BTDS) and validated with independent data set (BMIDS).

Other other side the model achieved 98.97% accuracy, 99.89% specificity, 99.39% sensitivity, 98.89% Precision, 99.40% MCC, and 99.30% F1-score when trained with augmented data set (BTDS) and validated with independent data set (BMIDS). Hence, from experimental results we observed that model predictive and generalization capability improved when trained and validated with independent data sets.

**Table 2:** CNN model performance with cross data set.

Data set	Parameters		Assessment metrics					
	Optimizer	LR	Acc (%)	Sp (%)	Sn (%)	Pr (%)	MCC (%)	F1-S (%)
Original	SGD	0.0001	97.96	99.00	97.30	98.18	98.00	99.02
Augmented	–	–	98.97	99.89	99.39	98.89	99.40	99.30

#### Results of the transfer learning models (ResNet-50, VGG-16, Inception V3, DenseNet201, Xception, and MobilleNet) on original and augmented data sets

The performances of transfer learning (ResNet-50, VGG-16, Inception V3, DenseNet201, Xception, and MobilleNet) models have checked on original and augmented data sets. Theses models have configured other essential hyper-parameters such as optimizer SGD with learning rate 0.0001, the number of epoch 100, batch size 120. The input image size $264\times 264\times 1$ is used for training and evaluation of the proposed model. The (ResNet-50, VGG-16, Inception V3, DenseNet201, Xception, and MobilleNet) models are evaluated using different performance evaluation metric. The (ResNet-50, VGG-16, Inception V3, DenseNet201, Xception, and MobilleNet) models hyper-parameters values and the output of the experimental results have reported in Table 3.

**Table 3:** Transfer learning models predictive performance on original and augmented data sets.

Model	Data set	Space complexity	Time complexity	LR	Assessment metrics					
	–	25.6M	3.2 h	0.0001	Acc (%)	Sp (%)	Sn (%)	Pr (%)	MCC (%)	F1-S (%)
ResNet50	Original			0.0001	97.03	97.04	93.10	94.21	93.23	95.00
	Augmented			–	98.07	99.30	100.00	96.07	96.00	97.00
VGG-16	–	138.4M	3.02 h	–	94.77	96.30	94.67	93.43	91.90	96.61
	–			–	95.97	96.95	99.40	96.84	92.98	96.80
Inception V3	–	23.9M	3.96 h	–	93.23	96.89	95.00	96.08	95.56	97.87
	–			–	96.03	97.03	97.00	97.01	96.05	98.00
DenseNet201	–	20.2M	3.9 h	–	96.76	95.99	95.60	95.78	98.45	98.23
	–			–	97.43	97.78	99.30	96.61	98.44	98.89
Xception	–	22.9M	4.3 h	–	93.00	97.03	98.00	97.09	99.32	97.23
	–			–	95.60	98.98	96.00	98.04	99.98	98.00
MobilleNet	–	4.3M	2.6 h	–	96.76	98.09	99.50	95.98	96.64	98.23
	–			–	97.87	99.00	94.56	96.23	98.45	97.89

The space complexity of each model is the number of trainable parameters. M = Million. The space complexity increases with increasing number of trainable parameters. The time complexity is the training time (in hours) of the models.

Table 3 show that the ResNet-50 model obtained 97.03% accuracy, 97.04% specificity, 93.10% sensitivity, 94.21% Precision, 93.23% MCC, and 95.00% F1-score respectively on original brain tumor data set. The 95.30% accuracy show that the ResNet-50 model accurately classifies the three classes of brain tumors (meningioma, glioma, and pictutitary). The 97.04% specificity shows that the ResNet-50 model is a highly suitable detecting model for healthy subjects recognition, while 93.10% sensitivity show that the model accurately detected the affected subjects.

The predictive Performance of transfer learning model ResNet-50 very high when model trained and evaluated with augmented data set. According to Table 3 the transfer learning model ResNet-50 obtained 98.07% accuracy, 99.30% specificity, 100.00% sensitivity, 96.07% precision, 96.00% MCC, and 97.00% F1-S, when trained and evaluated on augmented data set.

The VGG-16 model with original and augmented data sets obtained 94.77% accuracy, 96.30% specificity, 94.67% sensitivity, 93.43% precision, 91.90% MCC, 96.61% F1-S, and 95.97% accuracy, 96.95% specificity, 99.40% sensitivity, 96.84% precision, 92.98% MCC, and 96.80% F1-S respectively.

Inception V3 obtained 93.23% accuracy, 96.89% specificity, 95.00% sensitivity, 96.08% precision, 95.56% MCC, and 97.87% F1-s, with original data set. While on augmented data set Inception V3 obtained 96.03% accuracy, 97.03% specificity, 97.00% sensitivity, 97.01% precision, 96.05% MCC, 98.00% F1-S. DenseNet201 model obtained 96.76% accuracy on original data set and increase it 97.43% accuracy with augmented data set.

The Xception model with original data set achieved 93.00% accuracy, 97.03% specificity, 98.00% sensitivity, 97.09% precision, 99.32% MCC, 97.23% F1-S and obtained 95.60% accuracy, 98.98% specificity, 96.00% sensitivity, 98.04% precision, 99.98% MCC, and 98.00% F1-S with augmented data set. MobilleNet model obtained 96.76% accuracy with original data set and 97.87% with augmented data set. Among all models the ResNet-50 model performance in terms of accuracy is high with augmented data set. The model improved accuracy from 95.30 to 98.07% with data augmentation. The other evaluation metrics values also improved with data augmentation. From the experimental results, we concluded that the data augmentation process increased the training of ResNet-50 and model effectively classified the brain tumor types.

#### Results of the integrated frameworks (ResNet-50-CNN, VGG-16-CNN, Inception V3-CNN, DenseNet201-CNN, Xception-CNN, and MobilleNet-CNN) on original and augmented data sets

The integrated frameworks (ResNet-50-CNN, VGG-16-CNN, Inception V3-CNN, DenseNet201-CNN, Xception-CNN, and MobilleNet-CNN) performances have checked on original and augmented data sets. Furthermore, we have incorporated the TL ResNet-50, VGG-16, Inception V3, DenseNet201, Xception, and MobilleNet CNN architectures with imageNet data set to generate high weights and then transferred trained parameters weights of these pre trained models to the CNN model individually for effective training of CNN model. For fine-tuning of the CNN model, the brain tumors original and augmented data sets have used for final classification. The models have configured with concern hyper-parameters such as optimizer SGD with learning rate 0.0001, the number of epoch 100, batch size 120. The proposed framework performance has been evaluated employing various matrices. The input image size $264\times 264\times 1$ has been used for training and evaluation of the proposed model. All these hyper-parameters values and the output of the experimental results of (ResNet-50-CNN, VGG-16-CNN, Inception V3-CNN, DenseNet201-CNN, Xception-CNN, and MobilleNet-CNN) models have reported in Table 4.

Table 4 presented that the ResNet50-CNN model obtained 99.10% accuracy, 100.00% specificity, 89.60% sensitivity, 98.75% Precision, 98.66% MCC, and 99.5% F1-score respectively on original brain tumor data set. The 99.10% accuracy demonstrated that the architecture accurately classifies the three classes of brain tumors (meningioma, glioma, and pictutitary). The 100% specificity shows that the Proposed model is a highly suitable detecting model for healthy subjects recognition, while 89.60% sensitivity presents that the model significantly detected the affected subjects.

On the other hand, the model obtained very high performance when it trained and evaluated on the augmented data set. The integrated CNN and transfer learning model (ResNet-50-CNN) obtained 99.90% accuracy, 99.08% specificity, 96.13% sensitivity, and 99.10% MCC when trained and evaluated on augmented data set.

The VGG-16-CNN model with original and augmented data sets obtained 96.78% accuracy, 99.23% specificity, 95.00% sensitivity, 96.99% precision, 98.93% MCC, 97.98% F1-S, and 97.88% accuracy, 98.00% specificity, 100.00% sensitivity, 96.98% precision, 98.79% MCC, and 99.00% F1-S respectively.

Inception V3-CNN model obtained 97.00% accuracy, 99.00% specificity, 99.87% sensitivity, 98.92% precision, 95.76% MCC, 98.09% F1-S with original data set. While on augmented data set Inception V3 obtained 98.02% accuracy, 100.00% specificity, 98.67% sensitivity, 97.56% precision, 99.00% MCC, and 97.30% F1-S.

DenseNet201-CNN model obtained 97.00% accuracy on original data set and increase it 97.90% accuracy with augmented data set. Hence, the integrated model DenseNet201-CNN improved accuracy 97.00–97.90% = 0.90% with data augmentation process.

The Xception-CNN model with original data set achieved 98.20% accuracy, 98.88% specificity, 97.40% sensitivity, 99.00% precision, 99.10% MCC, 98.65% F1-S, and obtained 98.97% accuracy, 99.00% specificity, 98.60% sensitivity, 97.24% precision, 97.99% MCC, 99.30% F1-S with augmented data set. MobilleNet-CNN model obtained 98.08% accuracy with original data set and 98.56% with augmented data set. The improved accuracy 98.08% to 98.56% when model fine tuned with augmented data set.

**Figure 4:**
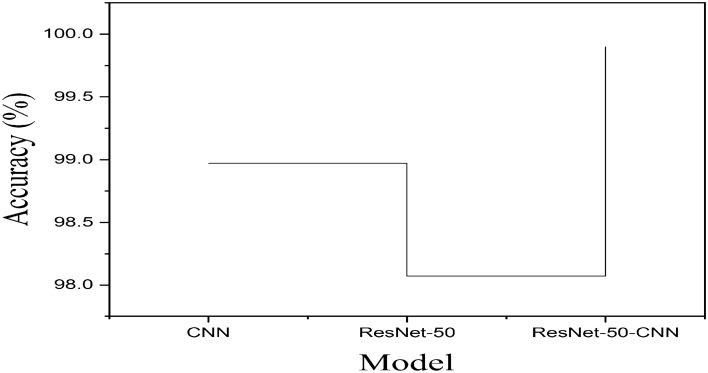
CNN, ResNet-50 and Integrated (ResNet-50-CNN) models accuracy comparison with augmented Brain tumor data set. The CNN model obtained accuracy’s with augmented data is 98.97%, while ResNet-50 obtained 96.07% and Integrated model ResNet-CNN obtained high predictive accuracy 99.90% with augmented data. Thus, the proposed integrated ResNet-CNN model is suitable for effective classification of brain tumors and could assist clinical professionals to diagnosis brain cancer accurately and efficiently. Due to the high performance of proposed ResNet-CNN method we recommend it for diagnosis of brain cancer in IoT-healthcare.

From above anlaysis we conculded that among all the ResNet-50-CNN, VGG-16-CNN, Inception V3-CNN, DenseNet201-CNN, Xception-CNN, and MobilleNet-CNN, the predictive performance of ResNet-50-CNN model is high in terms of accuracy. The accuracy of the model improved from 99.10 to 99.90% which is illustrated the importance of the data augmentation and transfer learning process. Hence we concluded that the ResNet-50-CNN model effectively classify the brain tumor types. The high accuracy of the proposed integrated diagnosis framework might be due to the suitable architecture of the model and proper fitting of essential parameters of the model and data augmentation. In addition, the proposed integrated model (ResNet-50-CNN) accuracy has compared with CNN model and transfer learning ResNet-50 model in Table 5 on augmented data set and graphically shown in Fig. 4.

**Table 4:** Integrated frameworks (ResNet-50-CNN, VGG-16-CNN, Inception V3-CNN, DenseNet201-CNN, Xception-CNN, and MobilleNet-CNN) performance on original and augmented data sets.

Model	Data set	Space complexity	Time complexity	LR	Assessment metrics					
	–	25.6M	3.3h	0.0001	Acc(%)	Sp(%)	Sn(%)	Pr(%)	MCC(%)	F1-S(%)
ResNet50-CNN	Original			0.0001	99.10	100.00	89.60	98.75	98.66	99.5
	Augmented			–	99.90	99.08	96.13	99.22	99.10	99.43
VGG-16-CNN	–	138.4M	3.21 h	–	96.78	99.23	95.00	96.99	98.93	97.98
	–			–	97.88	98.00	100.00	96.98	98.79	99.00
Inception V3-CNN	–	23.9M	4.13 h	–	97.00	99.00	99.87	98.92	95.76	98.09
	–			–	98.02	100.00	98.67	97.56	99.00	97.30
DenseNet201-CNB	–	20.2M	4.06 h	–	97.00	99.99	98.11	99.12	97.98	99.09
	–			–	97.90	100.00	97.45	95.68	99.03	99.32
Xception-CNN	–	22.9M	4.55 h	–	98.20	98.88	97.40	99.00	99.10	98.65
	–			–	98.97	99.00	98.60	97.24	97.99	99.30
MobilleNet-CNN	–	4.3M	2.7 h	–	98.08	99.43	93.20	99.12	97.65	93.231
	–			–	98.56	100.00	99.76	93.98	99.32	99.05

The space complexity of each model is the number of trainable parameters. M = Million. The space complexity increases with increasing number of trainable parameters. The time complexity is the training time (in hours) of the models.

**Table 5:** Accuracy of CNN, ResNet-50 and ResNet-50-CNN on augmented data.

CNN (Acc%)	ResNet-50 (Acc%)	ResNet-50-CNN (Acc%)
98.97	98.07	99.90

#### Accuracy comparison of the proposed (ResNet-CNN) model with state of-the-art models

**Figure 5:**
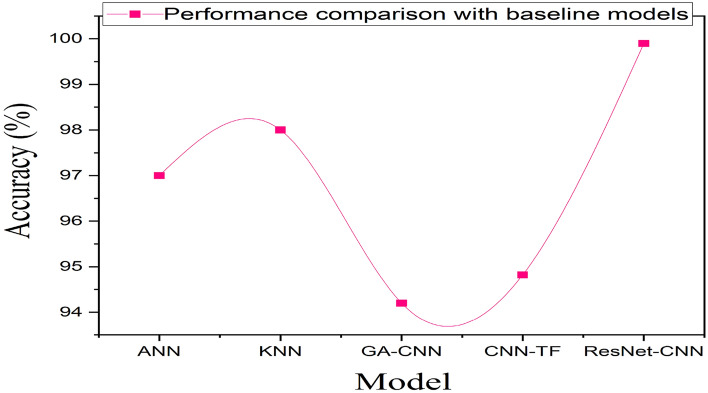
ResNet-CNN model performance comparison with baseline models show that our model predictive performance in terms of accuracy is high from baseline models. The ResNet-CNN model cloud accurately and efficiently classify the brain tumors and assist medical experts to interpret the images of brain tumors to diagnosis brain cancer.

We have compared our ResNet-50-CNN (ResNet-CNN) model performance in terms of accuracy with state-of-the-art methods in Table 6. Table 6 and Fig. 5 presented that proposed model obtained 99.89% accuracy, which is high as compared to state-of-the-art techniques. The high performance of the proposed method demonstrated that it is correctly classified brain tumors (meningioma, glioma, and pictutitary), and it can easily be deployed in IoT-health care for the classification of brain tumors.

**Table 6:** Comparison of ResNet-CNN model accuracy with previous models.

Model	Acc (%)	Ref	Space complexity	Time cmplexity
ANN and KNN	97, 98	^10^	$\mathscr {O}((nm + mk) + (n*d) )$	$\mathscr {O}((cwh) + (nd+Kn))$
GA-CNN	94.2	^12^	$$\mathscr {O}(cwh + 1)f$$	$$\mathscr {O}(f*u*m)$$
SVM and KNN	85, 88	^8^	$\mathscr {O}((n) + (n*d))$	$\mathscr {O}((n^2) + (nd+Kn))$
CNN-TF	94.82	^13^	$$\mathscr {O}(cwh + 1)f$$	$$\mathscr {O}(f*u*m)$$
Proposed method ResNet-CNN	99.90	2022	$$\mathscr {O}(cwh + 1)f$$	$$\mathscr {O}(f*u*m)$$

$$c=$$ the number of convolutional channels, $$h=$$ height of input, $$w=$$ width of input, $$f=$$ the convolutional kernel size, $$n=$$ the number data instances, $$k=$$ the number of output neurons, $$m=$$ the number of input neurons and $$d =$$ the dimension or feature of the input, $$K=$$ number of nearest neighbors, $$u=c*w*h$$.

#### Space and time complexity

Also, in Tables 3, 4, and 6, we present both the models space and complexity of the various proposed methods used in the prediction of Brain cancer. Since the proposed models are convolutional deep learning methods, the space complexities are analyzed in terms of the each model’s trainable parameters. For the time complexity, the model’s training time is used. It could be deduced from Table 3 that VGG-16 has the worst space complexity since its trainable parameter is 138.4 million, whiles MobileNet has the best space time complexity. Moreover for the time complexity, the Xception model has the worst time complexity because its training time is 4.3 h. Because of the difficulty of accessing the models of the competing methods in Table 4 , we could not experimentally analyze the complexity of the models in terms of algorithmic run-time. It is more likely that almost all the methods with the deep learning techniques, the convolutional neural networks will have a worse space and time complexity because of the significant number of parameters and matrix computation that come with the models’ architecture. Irrespective of the worst case time and space complexity, our proposed model has an accuracy performance gain as compared to all competing methods. The time complexity is the training time (in hours) of the models as reported in Tables 3, 4, and 6. The space and time complexity of our model are $$\mathscr {O}(cwh + 1)f$$ and $$\mathscr {O}(f*u*m)$$ respectively.

## Discussion

Brain Tumor Classification using MR images are critical in the detection of brain cancer In IoT healthcare systems. Artificial intelligence (AI) based computer automatic diagnostic systems (CAD) can effectively different diagnose diseases in IoT healthcare system. Deep learning techniques are widely used in CAD systems to diagnose critical diseases^32^, especially convolutional neural networks. The CNN model is mostly used for medical image classification^18,19^. The CNN model extracts deep features from image data, and these features played an important role in final image classification. For the diagnosis of brain cancer, various methods have been proposed by researchers using brain MR image data and deep learning models. However, these existing methods have lack of accuracy of diagnosis. In order to tackle this problem, a new method is necessary to diagnose the disease accurately and efficiently IoT healthcare systems.

In this study, we have proposed a CNN model for the accurate classification of brain tumor using Brain MR images. In the design of the proposed method, we have applied the deep learning CNN model for the classification of tumors meningioma, gLioma, and pituitary. The CNN model extracts more deep features from image data for final classification. To further improve the CNN model predictive capability, we have incorporated a transfer learning mechanism because, for proper training of the CNN architecture, the brain MR images data is insufficient. In transfer learning, we have used the well-known pre-trained models (ResNet-50, VGG-16, Inception V3, DenseNet201, Xception, and MobilleNet) with big imageNet data set to generate high parameters (weights). These generated weights of models individually transferred to CNN model for effective training. For the fine-tuning process, the model was trained with brain MR images data set. Also, the data augmentation method is employed to increase the data set size for effective training of the model. Furthermore, we have used held-out cross-validation and performance evaluation metrics. We also used cross data set for cehcking the propoed CNN model predictice performance.

According to Tables 2, 3, 4 and 6 the proposed method obtained high results as compared to baseline methods. The high performance of the proposed ResNet-CNN model might be due to the proper setting of model parameters such as learning rate, batch size, number of the epoch, and pre-processing, and data augmentation. We recommend the proposed method for meningioma, gLioma, and pituitary classification. Furthermore, the proposed method would be applied for diagnosis of a brain cancer in IoT-Healthcare systems easily.

## Conclusion

For accurate medical image classification, the CNN model is played a significant role, and in most CAD systems CNN model is used for the analysis of medical image data. In research study, we have proposed a deep learning-based diagnosis approach for brain tumor classification. In the proposed method, we have used a deep CNN model for the classification of tumor types Meningioma, Glioma, and Pituitary employing brain tumor MR images data. To enhance the predictive capability of the CNN model, we have incorporated transfer learning and data augmentation techniques. The experimental results show that the proposed integrated diagnosis framework ResNet-CNN has obtained 99.90% accuracy as compared to baseline methods. The high predictive outcomes of the proposed method might be due to the effective pre-processing of data and the adjustment of other parameters of the model such as numbers of layers, optimizer and activation functions, transfer learning, and data augmentation. Due to the high performance of the proposed ResNet-CNN model, it could be applicable for the classification of brain tumors and diagnosis of brain cancer in IoT-Healthcare. In the future, we will use other brain tumors datasets and other deep learning techniques to diagnose brain tumors.

## Data Availability

The data sets we used in this study are available on the kaggle machine learning repository at linked below: (1) Brain tumor dataset (https://www.kaggle.com/datasets/awsaf49/brain-tumor), and (2) Brain MRI Images for Brain Tumor Detection data set (https://www.kaggle.com/datasets/navoneel/brain-mri-images-for-brain-tumor-detection). All methods were performed in accordance with the relevant guidelines and regulations.
